# Bridging the gap: unveiling key links between autism and anxiety symptoms in autistic children and youth using a network analysis in pooled data from four countries

**DOI:** 10.1111/camh.70026

**Published:** 2025-09-02

**Authors:** Anat Zaidman‐Zait, Matthew J. Hollocks, Connor M. Kerns, Iliana Magiati, Alana J. McVey, Isabel M. Smith, Rachael Bedford, Teresa Bennett, Eric Duku, Stelios Georgiades, Annie Richard, Tracy Vaillancourt, Lonnie Zwaigenbaum, Antonio Hardan, Robin Libove, Jacqui Rodgers, Mikle South, Emily Simonoff, Amy Van Hecke, Mirko Uljarević, Peter Szatmari

**Affiliations:** ^1^ Tel‐Aviv University Tel‐Aviv Israel; ^2^ Institute of Psychiatry, Psychology & Neuroscience, King's College London London UK; ^3^ University of British Columbia Vancouver BC Canada; ^4^ The University of Western Australia Perth WA Australia; ^5^ Center for Behavioral Medicine Brookfield WI USA; ^6^ Dalhousie University and IWK Health Halifax NS Canada; ^7^ McMaster University Hamilton ON Canada; ^8^ University of Ottawa Ottawa ON Canada; ^9^ University of Alberta Edmonton AB Canada; ^10^ Stanford University Stanford CA USA; ^11^ Newcastle University Newcastle UK; ^12^ Emory University Atlanta GA USA; ^13^ Marquette University Milwaukee WI USA; ^14^ Melbourne School of Psychological Sciences University of Melbourne Melbourne Vic. Australia; ^15^ Telethon Kids Institute, University of Western Australia Perth WA Australia; ^16^ The Hospital for Sick Children University of Toronto Toronto ON Canada

**Keywords:** Autism spectrum disorders, anxiety, symptomatology

## Abstract

**Background:**

Autistic children experience significantly higher rates of anxiety compared to nonautistic children. The precise relations between autism characteristics and anxiety symptoms remain unclear in this population. Previous work has explored associations at the domain level, which involve examining broad categories or clusters of symptoms, rather than the relationships between specific symptoms and/or individual characteristics. We addressed this gap by taking a network approach to understand the shared structure of autism characteristics and anxiety symptoms.

**Method:**

Data were pooled from five studies from Canada, Singapore, the UK, and the USA, totaling 623 autistic children (17% female sex; aged 6–18 years), for whom the parent‐report Spence Children's Anxiety Scale (SCAS‐P) was available. We derived two undirected regularized networks, first from the SCAS‐P items only, and then by adding autism characteristics pertaining to social communication, highly focused and repetitive behavior, and sensory hypersensitivity. From these models' metrics, we extracted nodes' predictability, key bridging nodes, and community detection.

**Results:**

The anxiety‐only network was highly connected and consisted of four key clusters: General Anxiety, Social Anxiety, Separation Anxiety, and Panic/Agoraphobia. These broadly aligned with the existing SCAS‐P structure based on DSM‐IV‐TR criteria. In the autism‐anxiety network, the structure of anxiety remained mostly stable, with autism features forming their own community. Preference for predictability (i.e., sameness) and sensory hypersensitivity were key nodes that linked autistic features and anxiety symptoms, primarily through generalized anxiety.

**Conclusion:**

This study identified some of the key characteristics that bridge the broadly independent structures of autism characteristics and anxiety symptoms. The findings are discussed in the context of guiding the assessment, prevention, and treatment of anxiety in autism.

**Key practitioner message**

**What is known?**

Autistic children and youth often experience heightened levels of anxiety compared to their neurotypical peers. While the co‐occurrence of autism and anxiety is well documented, the specific characteristics of autism that contribute to anxiety symptoms remain unclear.

There is a need to move beyond traditional approaches and adopt more complex theoretical and methodological frameworks to gain insights into the underlying structure of anxiety symptoms in the context of autism. Network analysis can address this need.

**What is new?**

Network analysis revealed that autism characteristics form a single community, separate from but connected to anxiety symptoms, supporting the notion that autism and anxiety are related yet structurally distinct conditions. Key bridging nodes between autistic characteristics (preference for predictability/sameness and sensory hypersensitivity) and anxiety symptoms, particularly panic/agoraphobia (involving somatic symptoms) and generalized anxiety symptoms, were identified. These findings suggest that these autistic characteristics are critical nodes that may mitigate the association between autism and anxiety and are likely transdiagnostic.

**What is significant for clinical practice?**

Findings offer key insights into potential intervention and support targets that might disrupt the link between specific autism characteristics and anxiety. Interventions should focus on intolerance of uncertainty in autism and accommodations or interventions (e.g., environmental modifications) to reduce sensory hypersensitivity. Additionally, proactive measures that autistic individuals can undertake to navigate environmental demands more effectively are essential. These insights are crucial for developing personalized care strategies and improving clinical outcomes for autistic individuals experiencing anxiety.

## Introduction

Autism spectrum disorder (hereafter autism) is a neurodevelopmental condition characterized by social communication differences and difficulties and highly focused, repetitive patterns of interests, behavior, and activities (in the Diagnostic and Statistical Manual of Mental Disorders (DSM‐5‐TR); American Psychiatric Association, 2022; or ICD‐11; World Health Organization, 2023). Anxiety is a highly common co‐occurring condition in autistic children and adolescents (youth hereafter), with 20%–50% experiencing significantly elevated levels of anxiety (Kerns, Rast, & Shattuck, 2020; Lai et al., 2019; van Steensel, Bögels, & Perrin, 2011), substantially higher than reported in neurotypical children (Kerns et al., 2020). A better understanding of the nature of this co‐occurrence is imperative given that anxiety disorders remain stable and persist over time and into adulthood (Hollocks, Lerh, Magiati, Meiser‐Stedman, & Brugha, 2019), substantially interfere with daily, social, and academic functioning (Adams & Emerson, 2021; Chang, Quan, & Wood, 2012), and have been associated with elevated depressive symptoms (Kerns et al., 2020) and lower quality of life (Adams, Clark, & Simpson, 2020) in autistic youth.

Anxiety disorders experienced by autistic individuals broadly conform with the DSM‐5‐TR criteria (Kerns & Kendall, 2012; Wood & Gadow, 2010). Yet, anxiety symptoms might be missed due to diagnostic overshadowing such that anxiety symptoms are misattributed to autism (Rosen, Mazefsky, Vasa, & Lerner, 2018; Wood & Gadow, 2010). For example, it may be difficult to disentangle whether avoiding social situations indicates social anxiety and/or a manifestation of autism features. Adding to this complexity, autism‐related stressors and autism characteristics might contribute to autism‐related anxiety (e.g., anxiety associated with change to routine, novelty, and uncommon fears relating to autistic passions and interests, and other social fears) (Adams, Young, Simpson, & Keen, 2019; Kerns et al., 2021; Lau et al., 2020). Notably, characteristics of autism, such as a preference for predictability/routine, sensory hypersensitivity, and autism‐related social challenges, have been hypothesized to underlie both DSM‐aligned and these distinct or idiosyncratic anxiety presentations that can occur in autistic individuals (Boulter, Freeston, South, & Rodgers, 2014; Kerns & Kendall, 2012).

Although anxiety is elevated in autistic individuals, findings on the associations between autism characteristics and anxiety are inconsistent. Some studies have found positive significant associations (Sukhodolsky et al., 2008; Uljarević et al., 2020), whereas others have reported nonsignificant associations (Dubin, Lieberman‐Betz, & Michele Lease, 2015; Simonoff et al., 2008). More consistent results have been reported for studies examining the relationships between anxiety and specific subdomains of the autism phenotype (e.g., preference for sameness and sensory processing differences) (Duvekot, Ende, Verhulst, & Greaves‐Lord, 2018; Rodgers, Glod, Connolly, & McConachie, 2012; Russell, Frost, & Ingersoll, 2019). Despite the suggested association between autism characteristics and elevated anxiety, research has been hampered by a focus on associations at a domain level. This neglects the potential importance of associations at the individual symptom level, both within and between conditions, and in turn discounts the potential influence that certain autism characteristics might have on other characteristics (e.g., the effects of sensory sensitivity upon an increased need for routine) and on anxiety symptoms. This may confound direct relations without fully characterizing the complexity of the links among autism and anxiety symptoms, preventing vital insights into the etiology of mental health problems in autistic people. The high degree of co‐occurrence between different anxiety symptoms and the ongoing debate on the exact nature of the relations between anxiety and autism raise unanswered questions about shared causal relationships/pathways and potential areas of overlap (Kerns & Kendall, 2012; Wood & Gadow, 2010).

Traditional statistical approaches, such as structural equation modeling, have examined relationships between autism characteristics and anxiety at the level of latent variables or broad constructs, commonly introducing prior assumptions about the directionality of effects. While informative, these methods may obscure direct interactions between individual symptoms, thereby reducing their ability to capture the complex and dynamic interplay of symptoms within and across domains. In contrast, a network analysis approach provides a more nuanced, data‐driven understanding of these interactions, highlighting the nuanced relationships between autism characteristics and anxiety symptoms.

Network analysis is a promising approach for fine‐grained characterization of these issues. Instead of referring to an artifact of the diagnostic system due to overlapping symptoms, network theory conceptualizes mental health conditions as complex dynamic systems of interacting features that directly and causally influence one another's emergence and maintenance (Borsboom, 2017). In this way, symptoms and/or traits may be interrelated, and distinct phenotypes (or diagnostic categories) may best be viewed as emerging from specific patterns of interactions among traits across development rather than from a distinct set of anxiety symptoms, per se. Thus, in autism, anxiety may arise from challenges related to autism characteristics (Adams et al., 2019; Kerns & Kendall, 2012; Lau et al., 2020) and autistic features may also become more prominent because of anxiety symptoms (Hallett, Ronald, Rijsdijk, & Happé, 2010).

Network analysis provides a visual representation of the overall network structure, including its symptoms/characteristics (i.e., nodes) and their unique positive or negative relations between each pair of nodes (edges) (Epskamp & Fried, 2018). In network analysis, the most central nodes (i.e., symptoms/characteristics that are most strongly associated with other symptoms) are identified (Borsboom, 2017). In addition, nodes acting as a'bridge' between disorders can be identified. These bridging nodes represent possible causal pathways for the co‐occurrence of symptoms or disorders (Jones, Ma, & McNally, 2021). Furthermore, network analysis can determine which nodes form distinct communities within the network and whether these communities correspond to common diagnostic models (Golino & Epskamp, 2017). While network analysis can reveal complex direct and indirect patterns of associations that can generate hypotheses about potential causal relationships, it does not establish causality. These hypotheses need to be tested using methods specifically designed for causal inference, such as randomized controlled trials (Huang, Susser, Rudolph, & Keyes, 2023).

Finally, the network approach accounts for heterogeneity in characteristics such as age, sex, and overall level of functioning, which may influence the structure of the network. For example, how strongly or closely different symptoms are connected may be related to one or more of these individual characteristics (van Borkulo et al., 2015). This is particularly important when investigating anxiety, as it is well established that the prevalence rates of individual symptoms and disorders vary between childhood and adolescence (Hollocks et al., 2023) Additionally, sex differences have also been reported (Bargiela, Steward, & Mandy, 2016). Accordingly, the network approach provides a more detailed and nuanced description of the structure of mental health symptoms in autism, while accounting for other related characteristics such as age, as well as a visual representation of the structure of anxiety symptoms.

The unresolved complexities of the high co‐occurrence of anxiety disorders with autism, the potential overlap between autism features and anxiety symptoms, and the heterogeneity within diagnostic categories suggest the need to consider a different perspective and level of analysis. Accordingly, we examined the network structure of parent‐reported anxiety symptoms in a large sample of autistic youth while accounting for heterogeneity due to age and cognitive/adaptive functioning. We aimed to: (1) investigate connections among anxiety symptoms across several anxiety disorders, determine which symptoms form distinct communities (i.e., clusters of closely related symptoms), and estimate symptoms' centrality; and (2) examine the unique associations between traditional (DSM‐related) anxiety symptoms and autistic core characteristics, and to identify bridging nodes (i.e., symptoms and/or characteristics) between autism and anxiety symptoms in a network model.

## Method

### Participants

We conducted a network analysis using pooled cross‐sectional data from four countries. To ensure comprehensive and transparent reporting, we followed the Strengthening of the Reporting of Observational Studies in Epidemiology (STROBE) checklist for cross‐sectional studies (see Table S3). Data were obtained from a multisite data sharing initiative (see Table 1 for more details). Overall, five studies that had available data relating to autism characteristics, anxiety, and cognitive/adaptive functioning in autistic youth were included in the current examination. These studies were conducted in the United Kingdom (32 participants), Singapore (235 participants), the United States (97 participants), and Canada (259 participants). The aggregated sample included 623 caregivers of autistic children (aged between 6 and 18 years). All children had received a confirmed clinical/professional diagnosis of autism based on formal diagnostic criteria (DSM‐IV‐TR or ICD‐10) at the time of data collection. Ethics approval for each included study was granted by the relevant institutional ethics committees.

**Table 1:** Participant characteristics for each dataset included in the current pooled database

Study	*N*	Sample description	Recruitment and inclusion–exclusion criteria
Darus (2021)	32	UK community sample, not help seeking for anxiety. Recruited from mainstream and special schools	Children with either ASD based on SRS and parent‐reported established community diagnoses
		7–17 years old (*M* = 11.53 months, *SD* = 36.73); 84.4% Male	
Magiati, Chan, Tan, and Poon (2014), Magiati et al. (2016)	235	Singapore community sample; Recruited from special schools	Children diagnosed with ASD, Autism/Autistic Disorder, Asperger's Syndrome, or Pervasive Developmental Disorder‐Not Otherwise Specified (PDD‐NOS) based on established community diagnoses by professionals
		5–17 years old (*M* = 124.35 months, *SD* = 35.35); 81.7% Male	
Parker et al. (2014)	54	Children with ASD recruited through the Autism and Developmental Disorders Research Registry and by flyers posted in the Autism and Developmental Disorders Clinic at Stanford University	Children with ASD who met criteria for ASD order on the Autism Diagnostic Observation Schedule (ADOS; Lord et al., 2000) and ADI‐R diagnostic criteria
		6–12 years old (*M* = 115.94 months, *SD* = 22.97); 81.5% Male	Participants were prepubertal, in good medical health, and willing to provide a blood sample. Participants were included if they had a full‐scale IQ >50 and no genetic conditions
Szatmari et al. (2015)	259	The sample was drawn from the Pathways in Autism Spectrum Disorder study, a longitudinal project tracking the developmental trajectories of an inception cohort of children diagnosed with autism across five sites across Canada	Participants met criteria for ASD on the Autism Diagnostic Observation Schedule (ADOS; Lord et al., 2000) and in the social and one other domain of the Autism Diagnostic Interview‐Revised (ADI‐R; Rutter, LeCouteur, & Lord, 2003). They also met DSM‐IV‐TR criteria (American Psychiatric Association, 2000) according to the judgment of clinicians with diagnostic expertise
		7–12 years old (*M* = 122.21 months, *SD* = 11.67); 81.5% Male	Exclusion criteria included known genetic or chromosomal abnormalities, neuromotor disorders, and severe hearing or vision impairments
Van Hecke et al. (2015)	43	Community sample recruited via in‐house waiting list for social skills treatment at University Autism Clinic (not selected for anxiety)	Children with either Autism, Asperger, or PDD‐NOS based on established community diagnosis by professional and meeting criteria for ASD on the ADOS‐G Module 4
		11–16 years old (*M* = 161.02 months, *SD* = 16.81); 83.7% Male	Participants were fluent in English, had no history of major adolescent mental illness (such as bipolar disorder, schizophrenia, or psychosis), had no hearing, visual, or physical impairments, were motivated to learn how to make and keep friends, and had a Verbal IQ > 70

### Measures

#### Anxiety

The parent‐report Spence Children's Anxiety Scale (SCAS‐P; Nauta et al., 2004) is a 38‐item anxiety measure for children aged 6–18 years. Items describe anxiety symptoms across anxiety disorders based on the DSM‐IV‐TR criteria (APA; American Psychiatric Association, 2013). The SCAS‐P comprises six subscales: panic/agoraphobia, separation anxiety, social anxiety, generalized anxiety, physical injury/specific phobias, and obsessive‐compulsive disorder. Rated by caregivers from 0 (*never*) to 3 (*always*), higher scores indicate higher levels of anxiety. In the current study, only the panic/agoraphobia, separation anxiety, social anxiety, and generalized anxiety scales were included. The physical injury subscale describing specific fears or phobias was not included because of the low Cronbach's alpha reported consistently in previous studies and its limited coverage of various phobias (Lau et al., 2020; Magiati et al., 2016; Nauta et al., 2004). In addition, because obsessive‐compulsive disorder is no longer considered an anxiety disorder in the DSM‐IV‐TR (APA, 2013), we also excluded this subscale.

#### Autism‐related characteristics

Autism characteristics were assessed using items that were common across two measures used in the included studies: the Social Responsiveness Scale (SRS; Constantino & Gruber, 2005; *n* = 388) and the 29‐item Developmental Behavior Checklist Autism Screening Algorithm (Brereton, Tonge, Mackinnon, & Einfeld, 2002) (DBC‐ASA; *n* = 235). Three items pertained to social interaction and/or communication difficulties (i.e., neurotypical norm‐referenced difficulties in responding to others' feelings, atypical eye contact, and difficulties interacting with peers). Four items pertained to highly focused and repetitive patterns of interests, behavior, and activities (i.e., repetitive hand and body movements, highly intense interests, adherence to routine/strong preference for sameness, highly focused interests), and two items were related to sensory differences (i.e., atypical sensory interests and sensory hypersensitivity). Harmonizing autism characteristics across these measures maximized the sample size, enabling us to align with recent recommendations for sample size in network analysis, specifically ensuring that the number of observations exceeds the number of parameters (Epskamp & Fried, 2018).

#### Cognitive and adaptive functioning

Standardized IQ and adaptive functioning scores were obtained from assessments conducted in each study included in the dataset. While pooling data from multiple studies increases statistical power, it also presents challenges, particularly when different studies use measures that assess related, but not identical, constructs of intellectual and adaptive functioning. To address these challenges, we adopted an approach previously discussed and employed in previous studies (Griffith et al., 2015; Magiati, Ozsivadjian, & Kerns, 2017; Spackman et al., 2022). Specifically, the standardized tests used across the five studies (see Table 1) all had a mean standard score of 100 and a standard deviation of 15. However, instead of using continuous standardized scores, we harmonized the data by creating an ordinal ‘approximate level of functioning’ classification scale. This scale ranged from ‘1’ (*severe‐profound intellectual disability, standard score < 40, per DSM criteria*) to ‘8’ (*superior functioning, standard score ≥ 120*), aligning with classifications based on the Wechsler scales and DSM‐5 intellectual disability categories.

### Data analysis

#### Data preprocessing

For the covariate analyses, we examined correlations between age, cognitive/adaptive functioning, and sex (i.e., possible covariates) and all items included in the network analysis (see Table S1). All network data were investigated to ensure that they met assumptions for inclusion in the network models, including assessing item distributions, variance, and topological overlap (Fried & Cramer, 2017). Topological overlap between node pairs (very high overlap between nodes/items) was assessed using the ‘goldbricker’ function in R (Jones, 2020) with a threshold of 25% (correlations between items have significantly different correlations with 25% of the other items), accepting a minimum correlation of .5.

Considering the optimal way to model highly skewed items in networks (Isvoranu & Epskamp, 2023), and in line with previous studies (Ong et al., 2021; Rhemtulla et al., 2016), items that demonstrated near‐zero variance were excluded (see Appendix S1). We assessed item informativeness, or the standard deviation of each item, to evaluate whether each symptom had enough variation to be able to contribute to the network analysis (Mullarkey, Marchetti, & Beevers, 2019).

#### Network estimation

Data analyses were conducted using R (version 4.0.3). We estimated two networks: the first included anxiety symptoms only, and the second included autism characteristics and anxiety symptoms together, including covariates. In network modeling, nodes represent individual variables (questionnaire items in the present study) and edges represent conditional associations between two nodes in the network (Epskamp & Fried, 2018). We estimated two undirected regularized networks using the bootnet package in R to examine relations among anxiety symptoms and, in a separate analysis, relations between anxiety symptoms and autism characteristics.

We used the Gaussian Graphical Model (GGM), which estimates regularized partial association networks between a set of nodes (i.e., conditional independence) (Epskamp & Fried, 2018). To address multiple testing and prevent false positive edges (i.e., an estimated nonzero edge that is not present), the GGM applies the graphical least absolute shrinkage and selection operator (LASSO). The LASSO regularization adds to the estimation process a penalty parameter (tuning hyperparameter) that shrinks small edge‐weight coefficients to zero. We used the default tuning hyperparameter value (gamma = .5) as the most conservative approach in network analysis (Isvoranu & Epskamp, 2023). To account for the use of ordinal data, we based our analyses on Spearman correlations using pairwise complete observations (Epskamp, Borsboom, & Fried, 2018). As implemented in the qgraph R‐package (Epskamp, Cramer, Waldorp, Schmittmann, & Borsboom, 2012), the visualization of networks employed the Fruchterman–Reingold plotting method, which ensures a clear presentation of network edges and clustering structures, preventing overlapping nodes and minimizing edge crossings.

#### Network inference

To examine nodes' centrality, we calculated each node's expected influence, which reflects the overall level of a node's involvement in the network. Expected influence is the sum of a node's edges, accounting for both positive and negative values of edges (Haslbeck & Waldorp, 2018). We also calculated each node's predictability. Node predictability is an absolute measure of the interconnectedness of a node; it quantifies how well a particular node can be predicted by all remaining nodes (Haslbeck & Fried, 2017).

#### Community detection

A community is understood as a set of nodes with relatively more edges among nodes inside the community itself and the rest of the network. To examine the community structure of the networks, we utilized the Spinglass algorithm (Reichardt & Bornholdt, 2006) along with the ComDetSpin procedure (Werner, 2018). Spinglass is widely adopted in the network literature (Briganti, Kempenaers, Braun, Fried, & Linkowski, 2018) while outlining which nodes tend to be grouped together across all iterations.

#### Bridge nodes

To identify specific nodes that act as pathways linking different communities of anxiety symptoms, as well as connecting autism to anxiety, we employed bridge expected influence. Bridge expected influence quantifies the number and strength of edges a node has with nodes in other communities, highlighting its role in intercommunity connectivity. Higher bridge expected influence values indicate that a given symptom has stronger relations with symptoms in other communities, thus acting as a critical bridge in the network. To identify bridging nodes, we used the 80th percentile cutoff on the scores of nodes' bridge expected influence (Jones et al., 2021).

#### Stability and accuracy

To obtain information on the accuracy of all parameters' estimates, we used the nonparametric bootstrap and case‐dropping samples bootstrapping routines (1000 iterations), where a coefficient of stability (CS) greater than .5 indicates strong stability and interpretability implemented in R‐package bootnet (Epskamp et al., 2018).

Given the limited number of autistic characteristics in the harmonized pooled dataset, we conducted a sensitivity analysis to incorporate a broader range of autism characteristics alongside anxiety symptoms. Specifically, we performed a network analysis on a subsample with data from the SRS, which comprised the larger portion of the sample (*N* = 387). For this analysis, we used the SRS‐Short Form, consisting of 16 of the 65 original SRS items (SRS‐SF; Sturm, Kuhfeld, Kasari, & McCracken, 2017), which has been validated for its specificity in assessing core autistic characteristics (Oppenheimer, Weisskopf, & Lyall, 2024; Patti et al., 2023; Sturm et al., 2017). Using the SRS‐SF helped address the impact of the larger sample size while still capturing a broader range of autistic characteristics. However, as this analysis did not fully align with optimal sample size recommendations (Epskamp & Fried, 2018), it is considered exploratory, and the findings should be interpreted with caution. Consequently, the results from this exploratory analysis are provided in the Appendix S1 (see Appendix S1 for data analysis coded).

## Results

### Descriptive analyses

Baseline demographic characteristics for the aggregated sample are listed in Table 2. Data preprocessing, means, and standard deviations of all individual SCAS‐P and autism items are reported in the Appendix S1. Regarding covariates, only age and cognitive/adaptive functioning were significantly correlated with certain items included in the network analysis (see Table S1).

**Table 2:** Demographics and descriptive statistics of study variables

Variables	
Age in years	*M* = 10.5; *SD* = 2.3
Sex assigned at birth	Male, *N* = 517 (83%)

Ethnic background	*N*	%
Caucasian/White	267	42.9
East Asian (e.g., China, Japan, Korean)	196	31.5
Asian	67	10.8
Black/Afro‐Caribbean	13	2.1
South‐East Asian (e.g., Cambodian, Indonesian, Vietnamese, Philippines)	10	1.6
Arab/West Asian (e.g., Armenian, Egyptian, Iranian, Lebanese, Moroccan)	9	1.4
South Asian (e.g., East Indian, Pakistani, Punjabi, Sri Lankan)	8	1.3
Mixed ethnic group	7	1.1
Latin‐American	6	1
Indigenous/Aboriginal People	3	0.5
Missing	37	5.9

Cognitive/Adaptive Functioning (scale 1–8)	*M* = 4.2; *SD* = 2.1	
Cognitive/Adaptive Functioning – Tests used	*N*	%
Scales of Independent Behavior–Revised (SIB‐R; Bruininks, Woodcock, Weatherman, & Hill, 1996)	233	37.6
Vineland Adaptive Behavior Scales – Second Edition (VABS‐II; Sparrow, Balla, & Cicchetti, 2005)	17	2.7
Wechsler Abbreviated Scale of Intelligence (WASI; Wechsler, 1999)	147	23.6
Wechsler Intelligence Scale for Children–Third Edition (WISC‐III; Wechsler, 1991)	82	13.2
Stanford‐Binet Intelligence Scales–Fifth Edition (SB5; Roid & Sampers, 2004)	53	8.5
Kaufman Brief Intelligence Test–Second Edition (KBIT‐2; Kaufman & Kaufman, 2004)	43	6.9
Merrill–Palmer–Revised Scales of Development (Roid & Sampers, 2004)	18	2.9
Leiter International Performance Scale, Third edition (Leiter‐3; Roid, Miller, Pomplun, & Koch, 2013)	17	2.7
Wechsler Preschool and Primary Scale of Intelligence, Fourth Edition (WPPSI; Wechsler, 2012)	10	1.6

SCAS included items (abbreviate)	*M*	*SD*
Item 1 – Worries about things	1.1	0.7
Item 3 – Funny feeling in his/her stomach	0.4	0.7
Item 4 – Complains of feeling afraid	0.7	0.6
Item 5 – Afraid to be on his/her own at home	0.9	1.1
Item 6 – Scared to take a test	0.6	0.8
Item 7 – Afraid to use public toilets	0.4	0.8
Item 8 – Worries about being away from parents	0.8	0.8
Item 9 – Afraid to make him/herself a fool in front of people	0.6	0.8
Item 10 – Worries to do badly at school	0.6	0.8
Item 11 – Worries that something awful happen to a family member	0.4	0.7
Item 14 – Scared to sleep on his/her own	0.6	0.9
Item 15 – Trouble going to school in the mornings	0.2	0.5
Item 18 – Complains heart beating really fast	0.2	0.5
Item 20 – Worries that something bad will happen to him/her	0.3	0.6
Item 22 – (S)he feels shaky	0.6	0.6
Item 26 – Worries what other people think of him/her	0.7	0.7
Item 27 – Crowded places	0.8	0.8
Item 28 – Child feels really scared for no reason at all	0.6	0.6
Item 31 – Feels afraid to talk in front of the class	0.5	0.8
Item 33 – Worries to suddenly get a scared feeling	0.2	0.5
Item 34 – Afraid of being in small, closed places	0.3	0.6
Item 38 – Feel scared to stay away from home overnight	0.5	0.9

Autistic characteristics (abbreviate)	*M*	*SD*
1. Abnormal eye contact	1.1	0.7
2. Interacting peers	1.1	0.8
3. Responding feeling	1.2	0.8
4. Repetitive movements	0.8	0.8
5 and 6. Restricted interest and obsessive interests	1.2	0.8
7. Routine sameness	1.1	0.8
8. Unusual sensory interests	0.8	0.8
9. Sensory hypersensitivity	1.1	0.8

### Anxiety symptoms network

**Figure 1:**
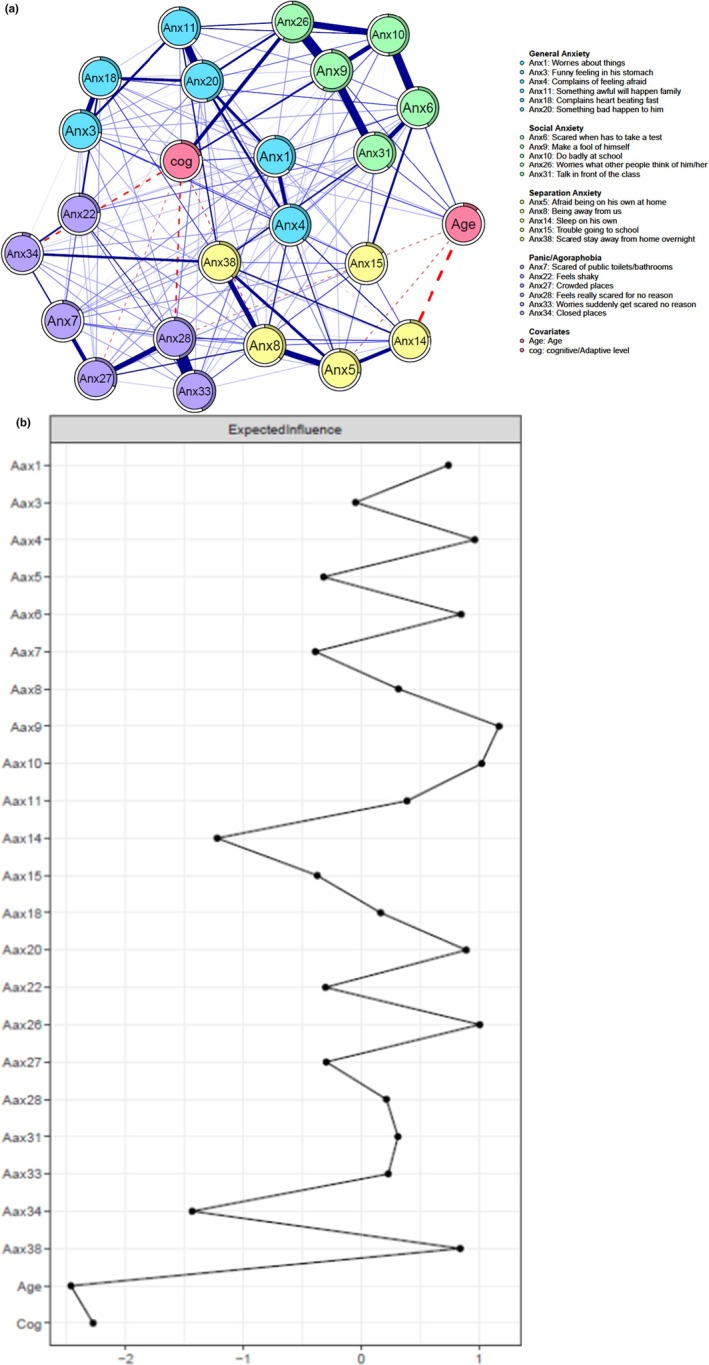
(A) Regularized partial correlation anxiety symptoms network. SCAS items are depicted as nodes, with edges connecting these nodes representing regularized partial Spearman correlations. Edge thickness represents the strength of the association. Blue (solid) edges indicate positive associations, and red (dashed) edges indicate negative associations. The colored part of the rings indicates the proportion of variance explained by all other nodes in the network. Anxiety symptoms are color‐coded based on community detection. The graph's layout is based on community detection and Lasso visualization. (B) Node expected influence of anxiety symptoms (individual SCAS items). The *x*‐axis represents the *z*‐standardized expected influence centrality values. The *y*‐axis indicates individual anxiety symptoms (based on SCAS items)

Upon inspecting the anxiety symptoms network (see Figure 1A) we found that all nodes were connected to at least one other node in the network. Of 276 possible edge weights, 165 (59.7%) were nonzero. To reiterate, an edge weight of 0.34 indicates that a person's score on item Anx28 is positively associated with their score on Anx33 after accounting for the covariance with all other nodes in the network. Notably, all edges between anxiety symptoms were estimated as positive, while several negative edges were observed between anxiety symptoms and the covariates (see Figure S1 for accuracy of edge weights). Results of the bootstrap routine to test the accuracy of the estimated edge weights indicated that many of the strongest edges had 95% CIs that did not overlap with zero, supporting the reliability of the edge weight estimates (Figure S1).

The strongest edges in the anxiety symptoms network were between two panic/agoraphobia symptoms, Anx28 (*feels really scared for no reason*) and Anx33 (*get scared for no reason*). In addition, there were relatively strong edges between social anxiety symptoms. Specifically, between Anx9 (*makes a fool of self*) and Anx26 (*worries what other people think of him*), as well as between Anx9 and Anx31 (*talks in front of the class*), and between Anx10 (*does badly at school*) and Anx6 (*feels scared when having to take a test*).

Additionally, there were relatively strong edges between social anxiety symptoms, such as between Anx9 (making a fool of oneself) and Anx26 (worries what other people think of them), as well as between Anx9 and Anx31 (talking in front of the class), and between Anx10 (doing badly at school) and Anx6 (scared to take a test). Results of the bootstrap routine to test the significance of the edge‐weight differences revealed that the above‐mentioned edges were significantly stronger than most other edges in the network (Figure S2).

The standardized expected influence centrality scores are displayed in Figure 1B. As can be seen in the figure, anxiety symptoms that exhibited the highest expected influence (EI = 1.10) included three social anxiety items (Anx9, Anx10, and Anx26) and a general anxiety symptom (Anx4 (*complains feeling afraid*)). Additionally, bootstrap difference tests revealed that these symptoms were significantly more central compared to approximately half of other symptoms (Figure S3). Stability analyses indicated adequate edge and expected influence centrality stability with coefficients of .59 and .52, respectively (Figure S4).

Nodes predictability estimates are displayed in Figure 1 (depicted by rings around each node). The average node predictability for the anxiety network was 32.9%. Among the social anxiety symptoms, Anx9, Anx10, and Anx26 exhibited high predictability ranging from 49% to 58%, indicating that these symptoms could be best explained by their associations with other nodes in the network. Similarly, Anx33 (*get scared for no reason*), Anx20 *(something bad will happen)*, and Anx28 (*feels really scared for no reason*), from the panic/agoraphobia and generalized anxiety subscales, showed moderate predictability ranging from 44% to 46%. Conversely, items originally assigned to the following SCAS‐P three subscales (panic/agoraphobia, separation, and social) including Anx34 (*afraid of closed places*, 6.7%), Anx15 (*trouble going to school*, 15.4%), and Anx7 (*afraid to use public toilets*, 15.7%) displayed relatively low predictability. This suggests that these symptoms may be independent of other factors within the network or influenced by factors not included in the current analysis.

The results of the Spinglass community detection analyses, along with the ComDetSpin procedure, are depicted in Figure 1A. The most common solution, observed in 68.5% of the 1000 iterations, identified four communities of anxiety symptoms, which align with the SCAS‐P division of items based on the DSM‐IV criteria: (1) General Anxiety, (2) Social Anxiety, (3) Separation Anxiety, and (4) Panic/Agoraphobia. Overall, three discrepancies emerged among the anxiety symptoms' communities and the SCAS‐P defined subscales. Firstly, Anx7 (*afraid to use public toilets*) was grouped within the Panic/Agoraphobia community (tapping mainly somatic and avoidance symptoms), although it is a SCAS‐P social anxiety subscale item. Secondly, Anx22 (*feels shaky*) was part of the General Anxiety community of symptoms, whereas SCAS‐P classified it as a panic symptom. Lastly, Anx11 (*something awful will happen to their family*) was included in the General Anxiety community of symptoms, whereas it was categorized by the SCAS‐P as a separation anxiety symptom.

Both covariates (i.e., age and cognitive/adaptive functioning) were primarily positively associated with the social anxiety community. It is worth noting that, in the second most common solution (28.8% of iterations), Anx15 (*trouble going to school*) did not belong to any anxiety disorder community. This suggests that Anx15 might oscillate across anxiety communities, indicating various underlying anxiety‐related reasons for school avoidance.

### Anxiety symptoms and autism characteristics network

**Figure 2:**
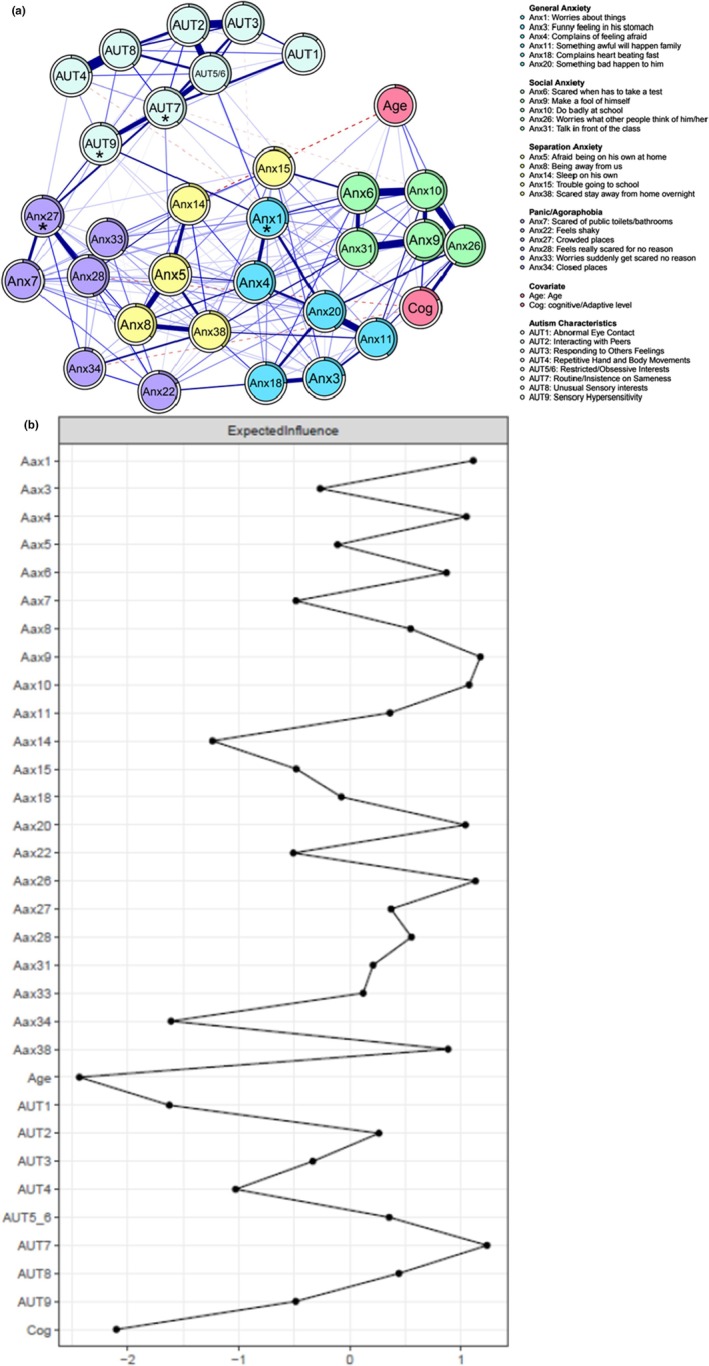
(A) Regularized partial correlation anxiety symptoms and autism characteristics network. SCAS items autism characteristics are depicted as nodes, with edges connecting these nodes representing regularized partial Spearman correlations. Edge thickness represents the strength of the association. Blue (solid) edges indicate positive associations, and red (dashed) edges indicate negative associations. The colored part of the rings indicates the proportion of variance explained by all other nodes in the network. (B) Node expected influence of anxiety symptoms and autism features. The *x*‐axis represents the z‐standardized expected influence centrality values. The *y*‐axis indicates individual anxiety symptoms and autism characteristics

In the combined network of anxiety symptoms and autism‐related characteristics (Figure 2A), of 496 possible edge weights, 203 (41%) were nonzero. After adding autism characteristics to the anxiety network, we found that the previously identified strongest edges were maintained, including the associations between the covariates (i.e., age and cognitive/adaptive functioning) that continued to primarily be associated with the social anxiety community. Additionally, relatively strong connections emerged between AUT4 (*repetitive movements*) and AUT8 (*sensory interests*). Results of the bootstrap routine testing confidence interval and significant edge weights' differences appear in the Figures S1B and S5. Mean node predictability remained similar at 31.5%, compared to 32.9% (see Figure 2B). The shared unique variances of the anxiety symptom nodes with all other nodes, including the autism characteristics nodes, were generally comparable to the previous network analysis that included only anxiety symptoms. Among the autism characteristics, the three nodes with the highest predictability values were AUT7 (*routine/insistence on sameness*), AUT8 (*sensory interests*), and AUT2 (*difficulties interacting with peers*).

Regarding nodes' centrality, nodes with the highest expected influence were consistent with those identified in the anxiety symptoms network alone (i.e., Anx9, Anx10, Anx26, and Anx4). Two other nodes also exhibited strong centrality: anxiety symptoms Anx1 (*worries about things*) and autistic trait item AUT7 (*routine/insistence on sameness*) (Figure 2B). The results of the bootstrap EI differences can be found in the Figure S6. Stability analyses indicated adequate edge and expected influence stability (.59 and .52 respectively; Figure S7).

Figure 2A displays the results of the Spinglass community detection. The most common solution, observed in 56.8% of the 1000 iterations, identified four communities. Two of these communities were consistent with the communities identified in the anxiety symptoms network, namely Separation Anxiety and Panic/Agoraphobia. However, the General Anxiety and Social Anxiety communities merged into a single joint community, along with the cognitive/adaptive functioning level. All autism characteristics formed a distinct community.

#### Bridge nodes

The highest bridging nodes between anxiety symptoms and autism characteristics included three anxiety symptoms: general worry (Anx1), fear of crowded places (Anx27), and concern about others' opinions (Anx26) as well as two autism‐related characteristics: sensory hypersensitivity (AUT9) and preference for predictability/sameness (AUT7). These nodes are marked with an asterisk in Figure 2A. Notably, the exploratory network analysis, which incorporated a broader range of autism characteristics using the SRS‐16 SF, indicated similar results (see Figure S8). The overall network structure remained consistent, with similar strong edges and comparable bridging nodes between anxiety and autism. Specifically, these bridging nodes included the same anxiety symptoms (i.e., Anx1, Anx26, Anx27) and comparable autism characteristics of sensory hypersensitivity and rigidity with routine, which reflects preference for predictability/sameness – and an additional characteristic (i.e., becoming upset when too much is happening). These parallels between the two network models support the robustness of our original analysis, which included fewer autism characteristics.

## Discussion

We investigated the network structure of anxiety symptoms as well as a combined network of anxiety symptoms and autism characteristics, in a large sample of autistic youth. Our novel findings advanced the field beyond traditional psychometric approaches such as factor analysis, which strives for simplicity in structure by excluding cross‐loading items. In utilizing a network approach, we embraced this complexity, allowing communities of symptoms to emerge while accounting for associations between them. This approach enhances our understanding of the relationships between autism and anxiety (i.e., nomological network; Cronbach & Meehl, 1955) and has important implications for understanding and treating the high rates of co‐occurring anxiety in autistic youth.

Community detection identified four distinct node communities that largely corresponded to the SCAS‐P subscales based on DSM criteria (i.e., General Anxiety, Social Anxiety, Separation Anxiety, and Panic/Agoraphobia). However, some discrepancies were observed, with certain anxiety symptoms displaying stronger connections to communities different from those defined by the SCAS‐P subscales. These discrepancies may reflect unique anxiety presentations in autistic individuals (Kerns et al., 2014, 2021) and, as such, may not always fit neatly into traditional diagnostic categories. Instead, they may be driven by different sources of fear, chronic worry, and heightened sensory sensitivity. For example, the fear of using public bathrooms—typically considered a characteristic of social anxiety—was instead connected to the ‘Panic/Agoraphobia’ items. This may be due to sensory experiences commonly encountered in public bathrooms, such as loud noises, strong odors, and unexpected sounds, which may directly trigger anxiety in autistic individuals, who are known to experience sensory hypersensitivity. Similarly, feeling shaky, typically considered a symptom of panic, and the fear that something bad will happen to family members, originally considered a symptom of Separation Anxiety, were predominantly connected to General Anxiety. These differences may reflect a more generalized state of heightened arousal and pervasive worry often observed in autistic individuals (Kerns & Kendall, 2012). Additionally, some anxiety symptoms may be harder to detect in autistic children with intellectual delays (Kerns et al., 2021), which could lead to a broader classification of anxiety as generalized anxiety.

Our finding that general anxiety symptoms act as key bridging symptoms, and that social anxiety nodes are highly central to the overall network of anxiety in autistic youth, aligns with research indicating that, alongside social anxiety, generalized anxiety is one of the most commonly diagnosed anxiety disorders in verbally fluent autistic youth and often co‐occurs with other anxiety disorders (Hollocks et al., 2023; Simonoff et al., 2008). Additionally, the finding that general anxiety acts as a bridging symptom (i.e., playing a crucial role in the connection and spread of anxiety symptoms across the network) highlights its central role in the interconnected nature of anxiety symptoms in a way that traditional analyses might not capture. This underscores how specific symptoms connect to nodes both within and outside their assigned communities, reflecting a network of components that influence each other (Borsboom, 2017).

In the combined network of anxiety symptoms and autism characteristics, the structure of the anxiety symptoms remained stable, with consistent relationships within and between the anxiety symptom communities. However, community detection indicated that social and generalized anxiety merged into a single community, a finding consistent with prior research in autism and more broadly, indicating that social and generalized anxiety commonly co‐occur and share many risk factors (Strawn, Lu, Peris, Levine, & Walkup, 2021; Sukhodolsky et al., 2008).

Additionally, community detection identified that autism characteristics formed a community, separate from but connected with anxiety. This finding supports prior research indicating that autism and anxiety are related but distinct conditions (Kerns et al., 2021; Kerns & Kendall, 2012; Vasa et al., 2023). It further suggests that the increased prevalence of DSM‐type anxiety disorders is associated with autism characteristics, while anxiety remains structurally distinct. These results imply that, at least for less idiosyncratic presentations of anxiety, autistic individuals may benefit from the psychological interventions designed for nonautistic people when suitably adapted to allow for full accessibility (Wood et al., 2020).

These findings help clarify the inconsistent findings of previous studies that explored the associations between anxiety and autism characteristics using subscale‐level data, rather than focusing on symptom‐level associations (Lau et al., 2020; Mingins, Tarver, Waite, Jones, & Surtees, 2021). Specifically, the finding that cognitive/adaptive functioning and age are more closely associated with the social anxiety community suggests that some effects may be driven by either cognitive or developmental level or may reflect differences in the likelihood of exposure to specific social experiences (Mylett et al., 2024).

The identification of bridging symptoms provides further insight into the co‐occurrence of autism and anxiety, highlighting shared pathways that may contribute to their co‐occurrence. Key bridging nodes included autistic characteristics (i.e., preference for predictability/sameness and sensory hypersensitivity) as well as anxiety symptoms (i.e., fear of being in crowded places, general worry, and concern about what others think). In terms of bridging autism characteristics, preference for predictability (i.e., sameness) was found to bridge autism to anxiety through its association with general worry about things. This finding aligns with prior research, which found that preference for sameness is positively associated with both intolerance of uncertainty (Boulter et al., 2014) and inflexible or repetitive thinking (Wigham, Rodgers, South, McConachie, & Freeston, 2015), both of which have been associated with anxiety in autistic individuals (Baribeau et al., 2021; Lei et al., 2022).

Another highly central autism bridging node was sensory hypersensitivity. Notably, sensory hypersensitivity was directly connected to other bridging nodes, including general worry and fear of crowded places. Each of these nodes was further linked to additional central bridging nodes (i.e., fear of public toilets and preference for predictability/sameness). In other words, sensory hypersensitivity established both direct and indirect pathways to all the highest bridging nodes, making it a central connecting node in the autism and anxiety symptoms network. Consistent with findings in a smaller sample of preschool children using a simplified network model (i.e., considering total scores for various types of anxiety and other constructs as nodes) (Vasa et al., 2023), insistence on sameness, intolerance of uncertainty, and sensory hypersensitivity were highly associated. These findings support the premise that these autistic characteristics are key nodes that may ameliorate the association between autism and anxiety. Importantly, these characteristics are likely to be transdiagnostic, and while more prominent in autistic youth, are likely to have application for anxiety in other children and adolescents (Carpenter et al., 2019). Indeed, given the link between sensory hypersensitivity and autonomic hyperarousal (Jung et al., 2021), one example might be to consider interventions that focus on supporting autistic individuals to regulate or improve their physiological state (such as heart rate variability biofeedback (Thoen, Alaerts, Prinsen, Steyaert, & Van Damme, 2023); or relaxation, which may be used with some autistic people).

These findings underscore the value of network analysis in clarifying the dynamic complex interactions between nodes in a system, revealing possible mechanisms through which anxiety and autism characteristics may be interconnected beyond the correlations between sensory hypersensitivity and anxiety shown in previous studies (Neil, Olsson, & Pellicano, 2016). First, heightened sensory sensitivity may increase worry, which in turn can lead to the activation of anxiety symptoms regarding sensory‐rich environments or stimuli (Mazefsky & Herrington, 2014). Second, sensory hypersensitivity may contribute to a preference for predictability and routine in autistic individuals, with situations introducing uncertainty or deviations from established patterns triggering worry and anxiety (Ng‐Cordell, Wardell, Stewardson, & Kerns, 2022; Sinclair, Oranje, Razak, Siegel, & Schmid, 2017).

Our findings support the development of interventions that may break the link between sensory experiences and anxiety. The identification of bridging symptoms provides key insights into possible intervention and support targets, which could be via either psychological or pharmacological means, or include approaches that target environmental factors (i.e., creation of positive environments). Some progress has been made in developing intervention approaches that focus on intolerance of uncertainty in autism, which aim to help autistic youth develop strategies for managing anxiety related to uncertainty (Rodgers et al., 2023). In addition, current interventions and strategies include supports and accommodations (i.e., environmental modifications) to reduce sensory hypersensitivity in addition to proactive measures that autistic individuals can undertake to navigate environmental demands more effectively and, in turn, ease sensory‐related distress, thus reducing the susceptibility for anxiety (Yuan et al., 2022).

This study has several key strengths, including a large sample of autistic youth and use of a well‐validated caregiver‐reported anxiety measure that maps onto DSM‐based anxiety disorders. The application of the network approach to understand the structure of the overlapping constructs of anxiety and autism with this level of detail is highly novel and a strength. Last, in the current examination we accounted for the effects of important correlates of anxiety (i.e., children's age and cognitive/adaptive functioning) among autistic children (Mingins et al., 2021).

There are several limitations to consider with respect to pooling datasets. To maximize power, we pooled data from several international datasets. Although this approach strengthens the analysis, it also introduces challenges, such as variations in the samples' demographic characteristics and potential sociocultural differences, including racial or ethnic identity. These factors, along with other site‐specific differences, could not be accounted for in the analysis due to sample characteristics and power constraints inherent in the complexity of network analyses. Additionally, data harmonization was required, including aligning cognitive and adaptive functioning scores across sites and measures. Furthermore, we were limited to a narrow range of items related to autism characteristics, which may have reduced our ability to detect associations with anxiety and diminished the specificity of our findings, and how they were related to, for example, specific sensory experiences.

Another limitation was the reliance on parent‐reported anxiety symptoms. While studies indicate good agreement levels between parent and child reports when assessing anxiety in autistic youth (Blakeley‐Smith, Reaven, Ridge, & Hepburn, 2012; Ozsivadjian, Hibberd, & Hollocks, 2014), discrepancies can occur (Ooi et al., 2016). Including self‐reports in future research would provide a more comprehensive understanding of anxiety experiences in autistic youth.

It is important to note that our sample includes a wide age range, which is significant given that rates of specific anxiety symptoms (e.g., reductions in separation anxiety) are known to change over time (Magiati et al., 2016; van Steensel et al., 2011). As discussed earlier, we addressed this by controlling for age in our analysis. Additionally, it is important to note that our sample predominantly consisted of individuals assigned male at birth, which may impact the generalizability of our findings to autistic individuals assigned female at birth. This warrants further investigation in future research.

Despite these limitations, our findings of a distinct autism symptom community primarily associated with anxiety through sensory sensitivity/intolerance of uncertainty to worry are an important advancement in the assessment and treatment of anxiety conditions in autistic children and youth. A key future direction would be to replicate and expand on the current findings by examining the networks between autistic characteristics and anxiety symptoms obtained through self‐reports, including a broader range of autistic characteristics. Importantly, as this study focused on DSM‐defined anxiety diagnoses, a crucial next step will be to leverage the methodological advantages of network analysis to explore: (i) the connections between DSM‐defined anxiety conditions and idiosyncratic experiences of anxiety in autism, and (ii) the relationships between autistic characteristics and non‐DSM autism‐related anxiety experiences.

## Funding information

This study was supported by the Canadian Institutes of Health Research (HDF‐70333, FDN 93621), Kids Brain Health Network (formerly NeuroDevNet), Autism Speaks, the Government of British Columbia, Alberta Innovates Health Solutions, and the Sinneave Family Foundation. The Magiati et al. (2016) study included in the dataset was supported by a start‐up grant to Dr. Magiati from the National University of Singapore, Faculty of Arts and Social Sciences, with which she was affiliated at the time the original study was completed.

## Ethical considerations

The research meets all the ethical guidelines. Informed consent was appropriately obtained. All authors have complied with ethical standards in the treatment of participants, and the research was approved by the Research Ethics Boards of all authors' universities, including the Stanford University Administrative Panel on Human Subjects in Medical Research (FWA00000935, eProtocol #14888‐2008); the National University of Singapore, Institutional Review Board (Ref Code 10‐440, approval number: NUS‐1255); the Marquette University Institutional Review Board; the Hamilton Integrated Research Ethics Board (REB; project number 04‐353, McMaster University); the local research ethics boards at all recruitment sites (University of British Columbia BREB, University of Alberta REO, McMaster University HiREB, McGill University REB, IWK Health Centre REB); and Newcastle University, Faculty of Medical Sciences Ethics Committee (0059_1/2013).

## Supporting information


**Appendix S1.** Supplementary: Methods.
**Figure S1.** Autism & anxiety symptoms network: Nonparametric bootstrapping results.
**Figure S2.** Anxiety symptoms network: Edge weights bootstrapped difference test.
**Figure S3.** Anxiety symptoms network: Expected influence bootstrapped difference test.
**Figure S4.** Anxiety symptoms network: Stability of expected influence centrality estimates assessed by case‐dropping subset bootstrap procedure.
**Figure S5.** Autism and anxiety symptoms network: Edge weights bootstrapped difference test.
**Figure S6.** Autism and anxiety symptoms network: Expected influence bootstrapped difference test.
**Figure S7.** Autism and anxiety symptoms network: Stability of the expected influence centrality assessed by case‐dropping subset bootstrap procedure.
**Figure S8.** Regularized partial correlation anxiety symptoms and autism characteristics network.
**Table S1.** Spearman correlations between potential covariates (sex, age, cognitive/adaptive functioning) and anxiety symptoms and autism characteristics.
**Table S2.** Summary of primary metrics and definitions in network analysis.
**Table S3.** STROBE statement – checklist of cross‐sectional studies.

## Data Availability

The data that support the findings of this study are available on request from the authors. The data are not publicly available due to privacy or ethical restrictions.
