# Supplementary material for: Bridging the gap: unveiling key links between autism and anxiety symptoms in autistic children and youth using a network analysis in pooled data from four countries
Source: Child Adolesc Ment Health. 2025 Sep 2;30(4):327–42. doi: 10.1111/camh.70026 (PMC12573069; doi:10.1111/camh.70026)
Supplement: Supplementary file 1 — Appendix S1. Supplementary: Methods. Figure S1. Autism & anxiety symptoms network: Nonparametric bootstrapping results. Figure S2. Anxiety symptoms network: Edge weights bootstrapped difference test. Figure S3. Anxiety symptoms network: Expected influence bootstrapped difference test. Figure S4. Anxiety symptoms network: Stability of expected influence centrality estimates assessed by case‐dropping subset bootstrap procedure. Figure S5. Autism and anxiety symptoms network: Edge weights bootstrapped difference test. Figure S6. Autism and anxiety symptoms network: Expected influence bootstrapped difference test. Figure S7. Autism and anxiety symptoms network: Stability of the expected influence centrality assessed by case‐dropping subset bootstrap procedure. Figure S8. Regularized partial correlation anxiety symptoms and autism characteristics network. Table S1. Spearman correlations between potential covariates (sex, age, cognitive/adaptive functioning) and anxiety symptoms and autism characteristics. Table S2. Summary of primary metrics and definitions in network analysis. Table S3. STROBE statement – checklist of cross‐sectional studies. [file CAMH-30-327-s001.docx]

**Supplementary Online Material**

Bridging the Gap: Unveiling Key Links Between Autism and Anxiety Symptoms in Autistic Children and Youth Using a Network Analysis in Pooled Data from Four Countries

**Methods:** Supplementary information

**List of Figures:**

Figure S1. Autism & anxiety symptoms network: Nonparametric bootstrapping results

Figure S2. Anxiety symptoms network: Edge weights bootstrapped difference test

Figure S3**.** Anxiety symptoms network: Expected influence bootstrapped difference test

Figure S4. Anxiety symptoms network: Stability of expected influence centrality estimates assessed by case-dropping subset bootstrap procedure

Figure S5. Autism & anxiety symptoms network: Edge weights bootstrapped difference test.

Figure S6. Autism & anxiety symptoms network: Expected influence bootstrapped difference test

Figure S7. Autism & anxiety symptoms network: Stability of the expected influence centrality assessed by case-dropping subset bootstrap procedure

Figure S8. Regularized partial correlation anxiety symptoms and autism characteristics network

**List of Tables:**

Table S1. Spearman correlations between potential covariates (sex, age, cognitive/adaptive functioning) and anxiety symptoms and autism characteristics

Table S2. Summary of primary metrics and definitions in network analysis

Table S3. STROBE statement - checklist of cross-sectional studies

**Supplementary: Methods**

*Data pre-processing.* Based on items informativeness and near zero variance, five SCAS items from the panic subscale were excluded (i.e., item #12 complains of suddenly feeling as if (s)he can't breathe) #30 (Suddenly becoming dizzy or faint), #32 (Complains of his heart suddenly starting to beat too quickly for no reason), #19 (starts to tremble or shake), and #25 (Feels scared if he has to travel in the car, or on a bus or train). No topological overlap across the SCAS items was identified (no pairs of nodes with 75% of correlations with other nodes being the same). Yet, goldbricker for the items assessing autism characteristics suggested an overlap between one pair of items ASD5 (Restricted Interests) and ASD6 (Obsessive Interests). The respective pair of items were combined into a single variable using principal component analysis (PCA) and were rescaled to their original Likert scale values to make variances comparable across all items (Terluin et al., 2016).

**Supplementary Figures**

| 1. Anxiety symptoms network | 1. Autism & Anxiety symptoms network |
| --- | --- |
| 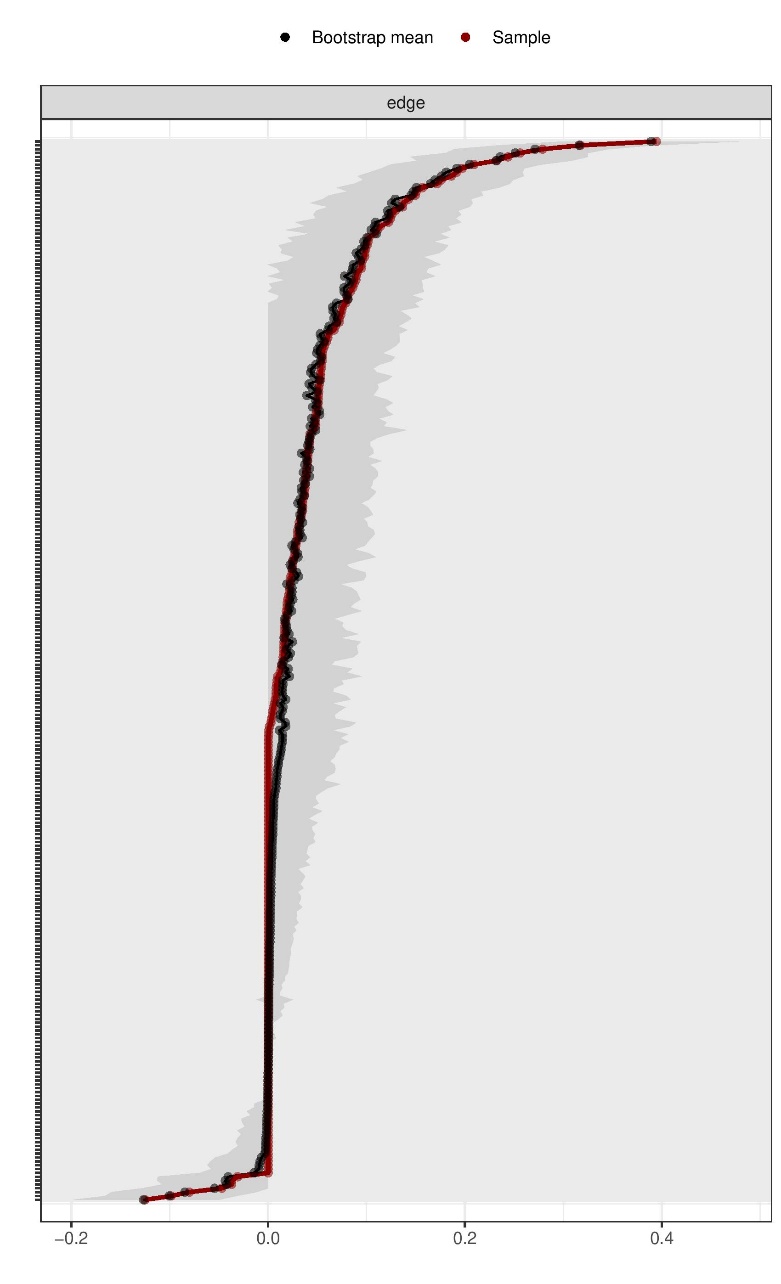 | 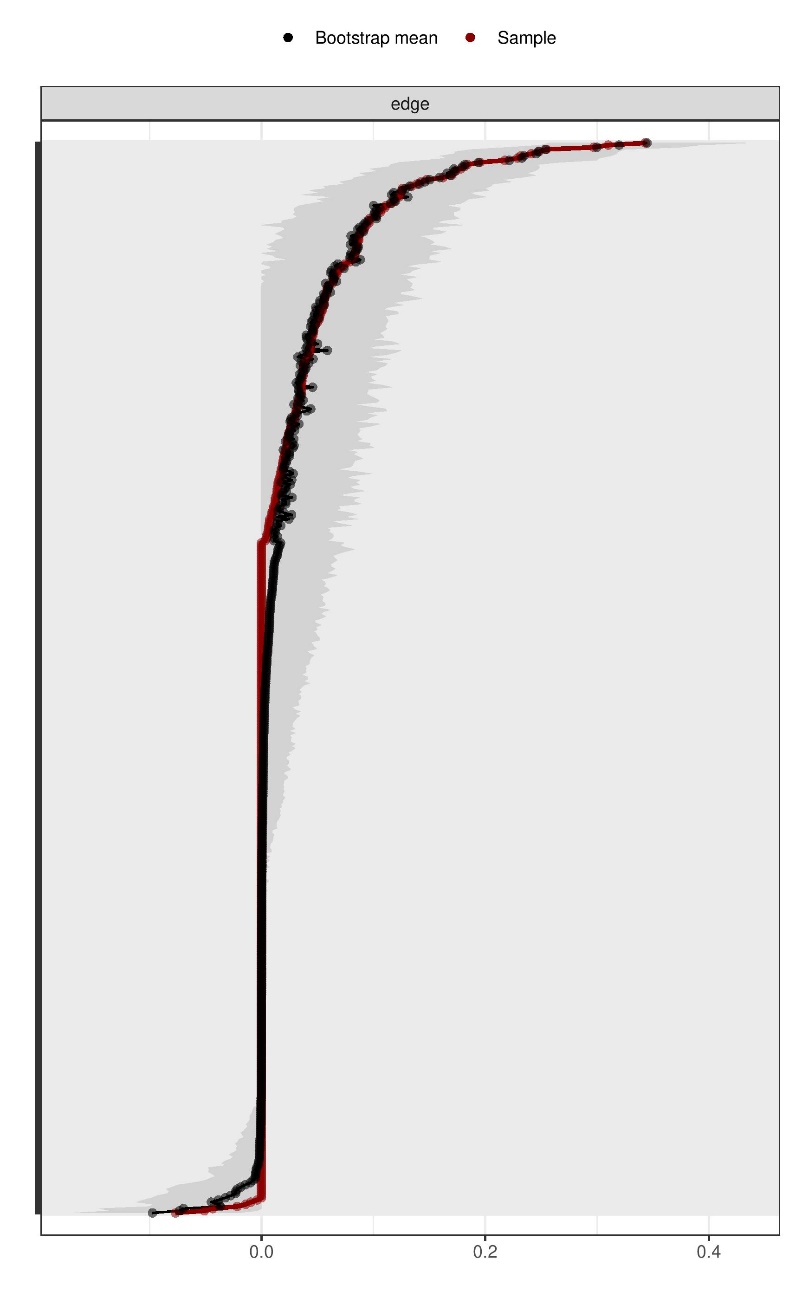 |
|  |  |

Figure S1. Autism & anxiety symptoms network: Nonparametric bootstrapping confidence intervals results with 1000 samples for anxiety symptoms network (A) and anxiety symptoms and autism characteristics network (B). The red line represents the estimated edge, while the dark area indicates the 95% bootstrap confidence interval. The smaller confidence intervals indicate more accurate edge estimates.


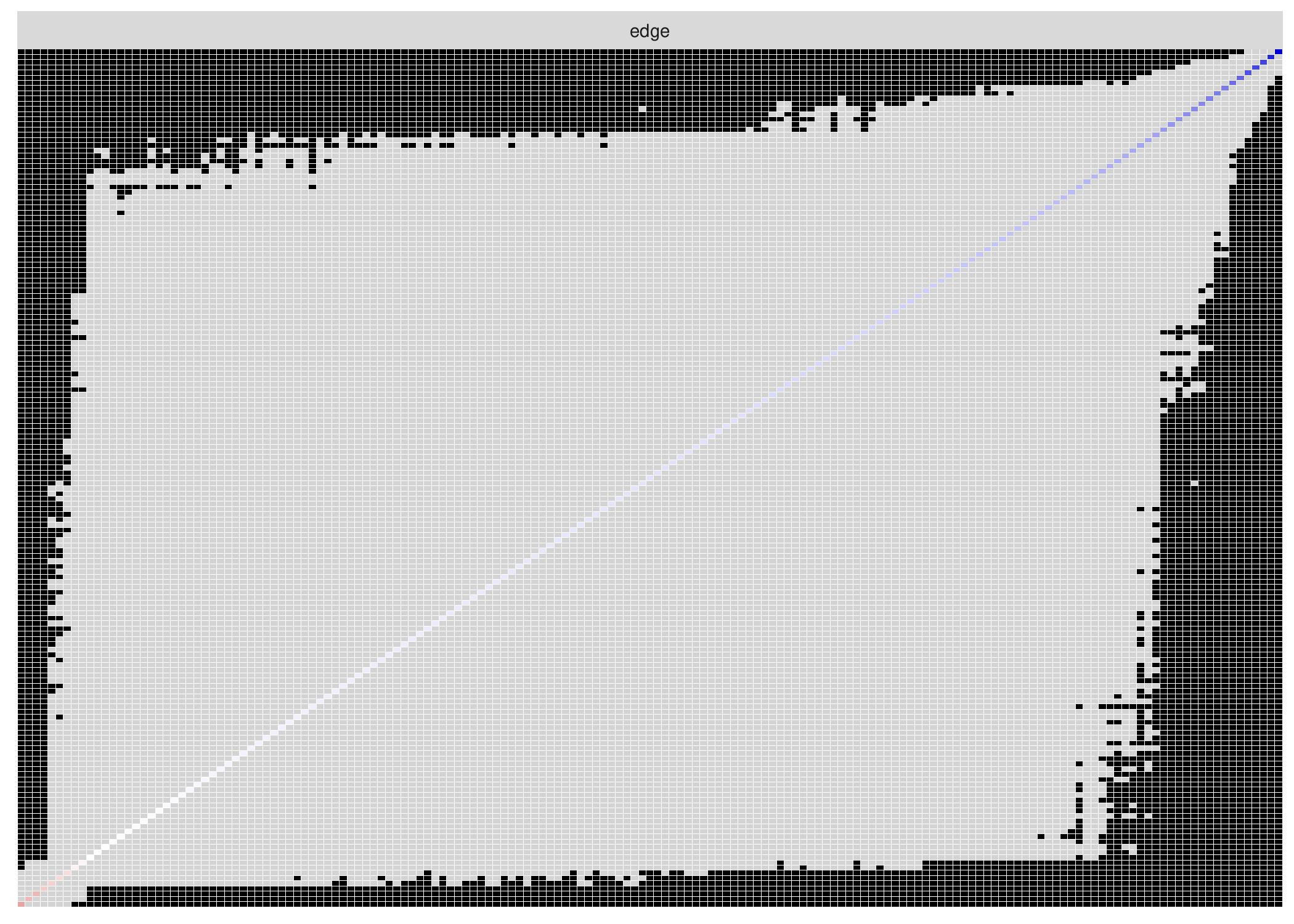


Figure S2. Anxiety symptoms network: Edge weights bootstrapped difference test. Grey boxes reflect no significant differences (α=0.05), and black boxes reflect significant differences.


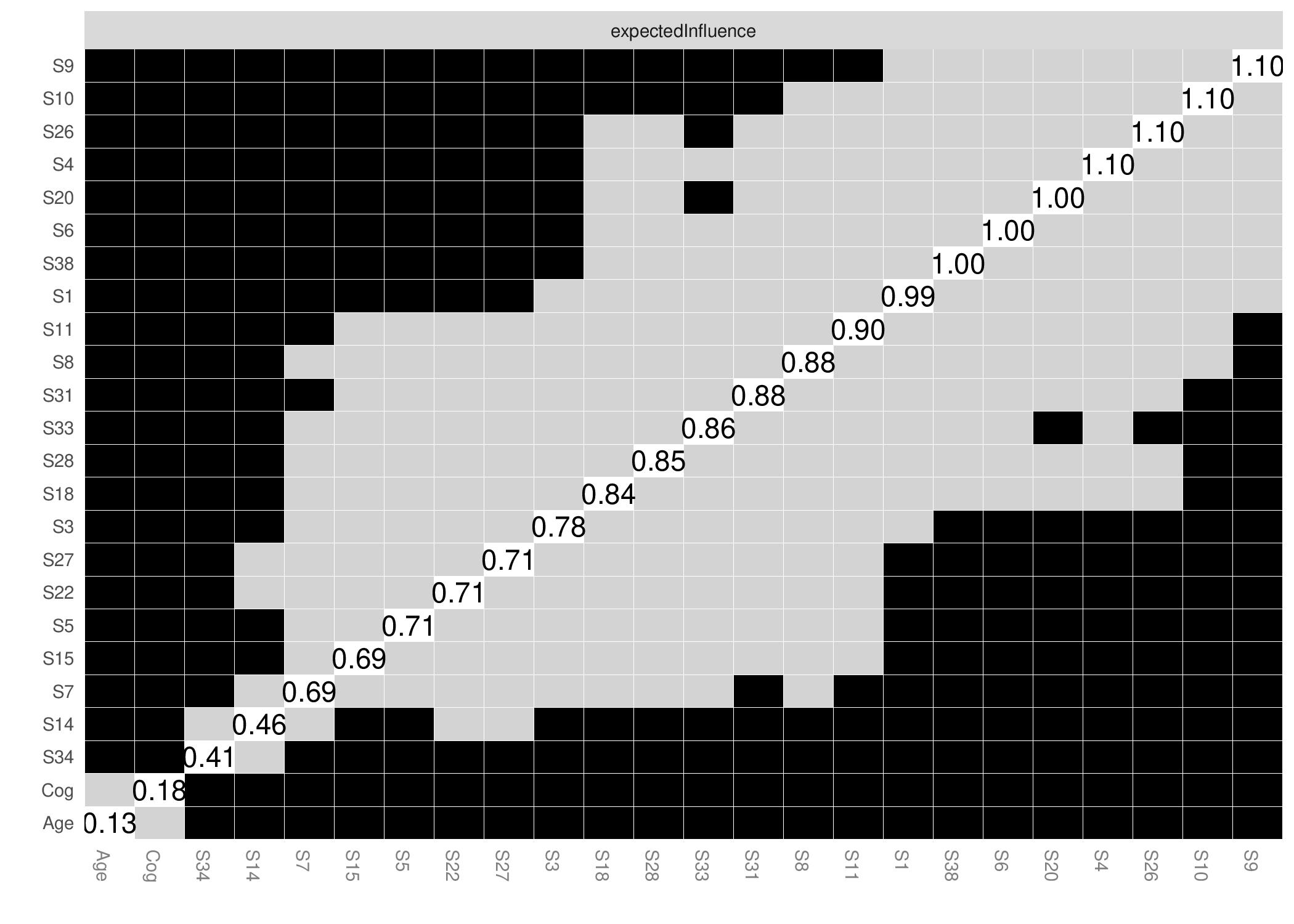


Figure S3**.** Anxiety symptoms network: Expected influence bootstrapped difference test (α=0.05). Grey boxes reflect no significant differences, and black boxes reflect significant differences.


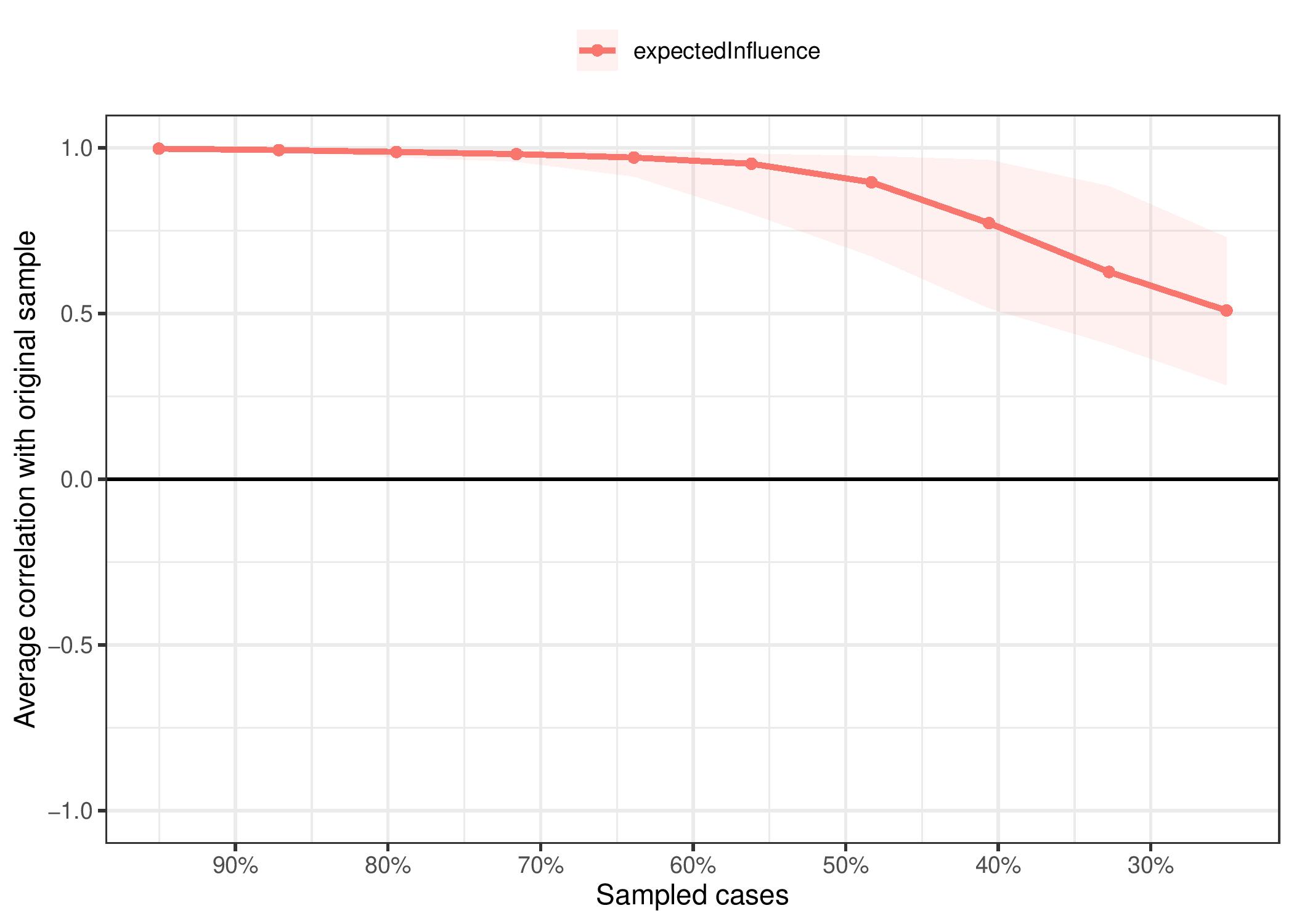


Figure S4. Anxiety symptoms network: Stability of expected influence centrality estimates

the expected influence centrality assessed by case-dropping subset bootstrap procedure. Decreasing portions of the participants were sampled, and the average correlations (with 95% confidence interval) are presented. After reducing the sample size to 50%, the average correlation with the results of the full population is preserved at a level of at least 0.52. This is an indication of the robustness of the network estimation.

**
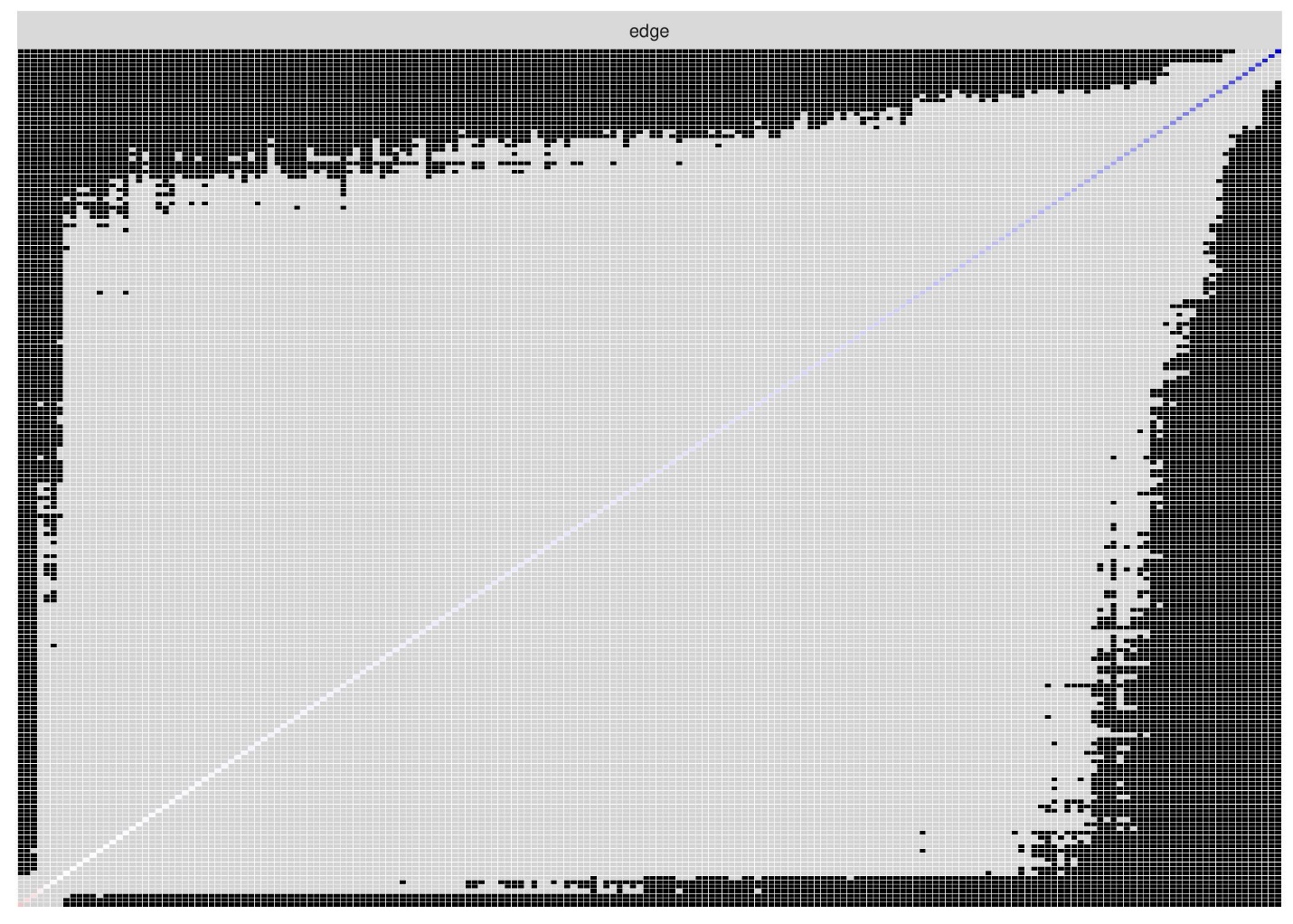
**

Figure S5. Autism & anxiety symptoms network: Edge weights bootstrapped difference test. Grey boxes reflect no significant differences (α=0.05), and black boxes reflect significant differences.


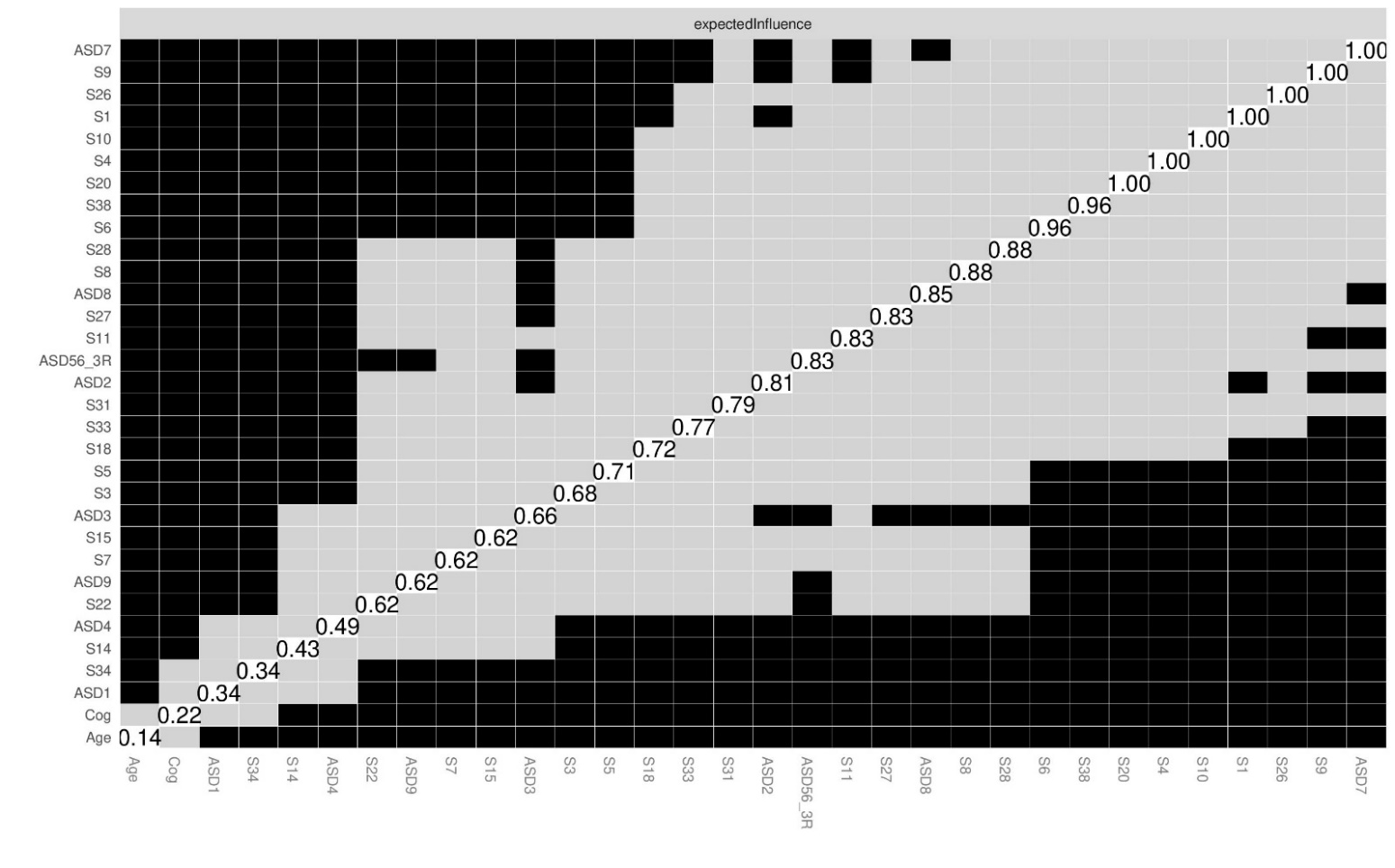
 Figure S6. Autism & anxiety symptoms network: Expected influence bootstrapped difference test. Black boxes indicate a significant difference in node expected influence centrality for each pairing, grey a non-significant difference


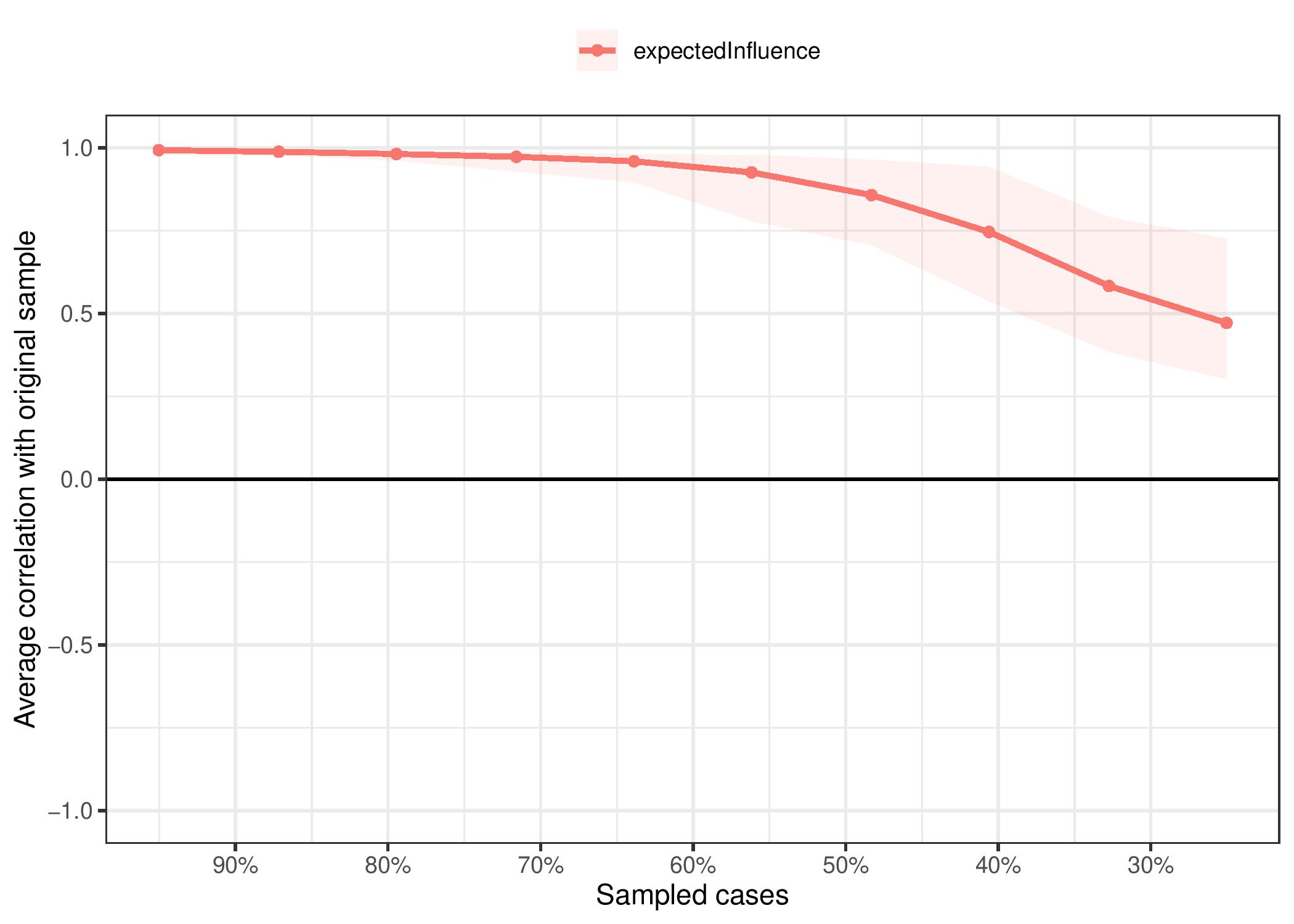


Figure S7. Autism & anxiety symptoms network: Stability of the expected influence centrality assessed by case-dropping subset bootstrap procedure. Decreasing portions of the participants were sampled, and the average correlations (with 95% confidence interval) are presented. After reducing the sample size to 50%, the average correlation with the results of the full population is preserved at a level of at least 0.52. This is an indication of the robustness of the network estimation.


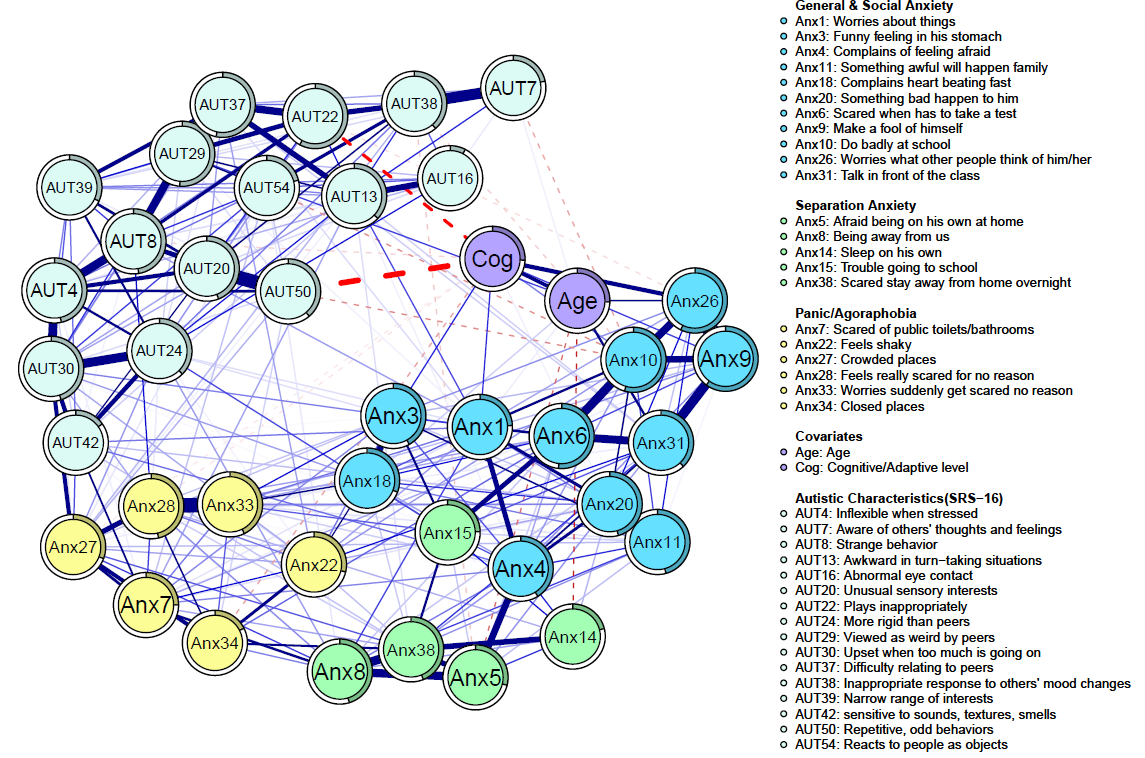


Figure S8. Regularized partial correlation anxiety symptoms and autism characteristics network. SCAS and SRS-SF items are depicted as nodes, with edges connecting these nodes representing regularized partial Spearman correlations. Edge thickness represents the strength of the association. Blue (solid) edges indicate positive associations, and red (dashed) edges indicate negative associations. The colored part of the rings indicates the proportion of variance explained by all other nodes in the network.

Table S1. Spearman correlations between potential covariates (sex, age, cognitive/adaptive functioning) and anxiety symptoms and autism characteristics

| Anxiety symptoms | Sex | Age | Cognitive |
| --- | --- | --- | --- |
| Anx1 | .01 | .20** | .24** |
| Anx3 | -.01 | .08* | .19** |
| Anx4 | .05 | .07 | .16** |
| Anx11 | .01 | .11** | .23** |
| Anx18 | -.03 | .14** | .16** |
| Anx20 | .00 | .13** | .18** |
| Anx6 | .03 | .20** | .13** |
| Anx9 | .02 | .15** | .33** |
| Anx10 | .02 | .25** | .30** |
| Anx26 | .07 | .25** | .37** |
| Anx31 | -.04 | .21** | .16** |
| Anx5 | .07 | -.11** | .05 |
| Anx8 | .03 | -.04 | -.10* |
| Anx14 | -.02 | -.19** | -.08* |
| Anx15 | .03 | .08* | .04 |
| Anx38 | .00 | .07 | .04 |
| Anx7 | .04 | -.02 | -.03 |
| Anx22 | .03 | .07 | -.05 |
| Anx27 | -.03 | -.01 | -.11** |
| Anx28 | .02 | -.10** | -.15** |
| Anx33 | .05 | .00 | -.03 |
| Anx34 | .00 | .01 | -.16** |
| Autism characteristics |  |  |  |
| AUT1 | -.06 | -.04 | -.11** |
| AUT2 | -.06 | .08 | .02 |
| AUT3 | .00 | .05 | .04 |
| AUT4 | -.04 | .00 | -.14** |
| AUT5 | -.06 | .12** | -.08* |
| AUT6 | -.05 | .09* | .10* |
| AUT7 | -.04 | .02 | -.04 |
| AUT8 | -.08 | .00 | -.07 |
| AUT9 | .01 | -.04 | -.04 |
| Sex |  | .05 | -.08 |
| Age |  |  | .18** |

Table S2. Summary of primary metrics and definitions in network analysis

| Metric | Definition |
| --- | --- |
| Network Structure | The structure of a network is characterized by the set of edges connecting its nodes. |
| Node | Observed variables in a dataset (e.g., questionnaire items in the present study) representing the variables of interest. |
| Edge | Conditional associations between two nodes in the network. Edges represent the strength of connections between nodes. |
| Fruchterman-Reingold Plotting Method | Visualization method used to ensure clear presentation of network edges and clustering structures, minimizing overlapping nodes and edge crossings. |
| Topological Overlap | Two nodes exhibit topological overlaps if they share the same relations with other nodes in the network. |
| Node Predictability | Quantifies how well a node can be predicted by all other nodes in the network. This absolute measure reflects a node's interconnectedness. |
| Centrality | A node's influence in terms of its direct and/or indirect connections to other nodes in the network. Higher values indicate more influential or central nodes. |
| Centrality: Node Expected Influence | A measure reflecting the strength and nature of a node's influence on other nodes in the network, accounting for both positive and negative edge values. |
| Community | Substructures within the network that may have distinct functions or characteristics. Communities are groups of nodes with dense internal connections and sparse external ones. |
| Bridge Centrality | Indicates nodes within a community that are most strongly connected to nodes in other communities. Calculated as the sum of edge weights connecting the node to other communities. |

(Epskamp & Fried, 2018; Haslbeck & Fried, 2017; Haslbeck & Waldorp, 2018; Jones et al., 2021; Reichardt & Bornholdt, 2006)

Table S3. STROBE statement - checklist of cross-sectional studies

|  | Item No. | Recommendation | Respected? | Section |
| --- | --- | --- | --- | --- |
| **Title and abstract** | 1 | (a) Indicate the study’s design with a commonly used term in the title or the abstract | Yes | Title & Abstract |
|  |  | (b) Provide in the abstract an informative and balanced summary of what was done and what was found | Yes | Abstract |
| **Introduction** |  |  |  |  |
| Background/rationale | 2 | Explain the scientific background and rationale for the investigation being reported | Yes | Introduction |
| Objectives | 3 | State specific objectives, including any prespecified hypotheses | Yes | A statement at the end of the introduction specifies the specific goals and objectives. |
| **Methods** |  |  |  |  |
| Study design | 4 | Present key elements of study design early in the paper | Yes | Study design is stated in the first section of Methods. |
| Setting | 5 | Describe the setting, locations, and relevant dates, including periods of recruitment, exposure, follow-up, and data collection | Yes | Described in the Methods & Supplement Table 1 |
| Participants | 6 | Cross-sectional study—Give the eligibility criteria, and the sources and methods of selection of participants | Yes | Methods: Participants & Table 1. & Supplement Table 1 |
| Variables | 7 | Clearly define all outcomes, exposures, predictors, potential confounders, and effect modifiers. Give diagnostic criteria, if applicable | Yes | Methods: Measures & Data analysis |
| Data sources/ measurement | 8 | For each variable of interest, give sources of data and details of methods of assessment (measurement). Describe comparability of assessment methods if there is more than one group | Yes | Methods: Data analysis |
|  |  |  |  |  |
| Bias | 9 | Describe any efforts to address potential sources of bias | Yes | We have carefully managed and addressed potential bias related to pooling datasets and harmonizing autism characteristic measures. These efforts are discussed across several sections of the manuscript, including data analysis, supplementary material, sensitivity analysis, and the discussion. |
| Study size | 10 | Explain how the study size was arrived at | Yes | We described the different studies included in the pooled dataset in detail. Additional information about these studies is provided in Supplementary Table 1 |
| Quantitative variables | 11 | Explain how quantitative variables were handled in the analyses. If applicable, describe which groupings were chosen and why | Yes | These are described in the method and data analysis sections |
| Statistical methods | 12 | (a) Describe all statistical methods, including those used to control for confounding | Yes | Methods: Data analysis |
|  |  | (b) Describe any methods used to examine subgroups and interactions |  | Not relevant |
|  |  | (c) Explain how missing data were addressed | Yes | Methods: Data analysis |
|  |  | (d) Cross-sectional study—If applicable, describe analytical methods taking account of sampling strategy | Not applicable | |
|  |  | (e) Describe any sensitivity analyses |  | Supplementary material: Sensitivity analysis |
| Results |  |  |  |  |
| Participants | 13* | (a) Report numbers of individuals at each stage of study |  | Not applicable |
|  |  | (b) Give reasons for non-participation at each stage |  | Not applicable |
|  |  | (c) Consider use of a flow diagram |  | Not applicable |
| Descriptive data | 14* | (a) Give characteristics of study participants (e.g. demographic, clinical, social) and information on exposures and potential confounders | Yes | Methods: Participants  Results: First paragraph & Table 1  Supplementary material: Table 2S |
|  |  | (b) Indicate number of participants with missing data for each variable of interest |  | Table 1 |
| Outcome data | 15 | Cross-sectional study—Report numbers of outcome events or summary measures | Yes | Not applicable |
| Main results | 16 | (a) Give unadjusted estimates and, if applicable, confounder-adjusted estimates and their precision (e.g., 95% confidence interval). | Yes | Results & supplementary material: Network accuracy & stability |
|  |  | (b) Report category boundaries when continuous variables were categorized | Yes | Not applicable |
|  |  | (c) If relevant, consider translating estimates of relative risk into absolute risk for a meaningful time period |  | Not applicable |
| Other analyses | 17 | Report other analyses done | Yes | Supplement: Exploratory analysis |
| Discussion |  |  |  |  |
| Key results | 18 | Summarize key results with reference to study objectives | Yes | Discussion |
| Limitations | 19 | Discuss limitations of the study, taking into account sources of potential bias or imprecision. Discuss both direction and magnitude of any potential bias | Yes | Limitations and future directions |
| Interpretation | 20 | Give a cautious overall interpretation of results considering objectives, limitations, multiplicity of analyses, results from similar studies, and other relevant evidence | Yes | Discussion |
| Generalizability | 21 | Discuss the generalizability (external validity) of the study results | Yes | Limitations and future directions |
| Other information |  |  |  |  |
| Funding | 22 | Give the source of funding and the role of the funders for the present study and, if applicable, for the original study on which the present article is based | Title page | |

- Link to data analysis code: https://github.com/AnatZZ/Code_SCAS
